# Artificial Intelligence and the Future of Cardiac Implantable Electronic Devices: Diagnostics, Monitoring, and Therapy

**DOI:** 10.3390/jcm14248824

**Published:** 2025-12-13

**Authors:** Ibrahim Antoun, Alkassem Alkhayer, Ahmed Abdelrazik, Mahmoud Eldesouky, Kaung Myat Thu, Harshil Dhutia, Riyaz Somani, G. André Ng

**Affiliations:** 1 Department of Cardiology, University Hospitals of Leicester NHS Trust, Glenfield Hospital, Leicester LE3 9QP, UK; ia277@leicester.ac.uk (I.A.); ahmed.abdelrazik@leicester.ac.uk (A.A.); mie7@leicester.ac.uk (M.E.); kaung.thu1@nhs.net (K.M.T.); h.dhutia@nhs.net (H.D.); riyaz.somani1@nhs.net (R.S.); 2 Department of Cardiovascular Sciences, Clinical Science Wing, University of Leicester, Glenfield Hospital, Leicester LE3 9QP, UK; 3 Department of Cardiology, Guys and St Thomas Hospital, London SE1 7EH, UK; alkassem.alkhayer92@gmail.com; 4 National Institute for Health Research Leicester Research Biomedical Centre, Leicester LE5 4PW, UK; 5 Leicester British Heart Foundation Centre of Research Excellence, Glenfield Hospital, Groby Road, Leicester LE3 9QP, UK

**Keywords:** implantable cardiac devices, machine learning, pacemaker, artificial intelligence, cardiac defibrillator

## Abstract

Cardiac implantable electronic devices (CIEDs) such as pacemakers, implantable cardioverter-defibrillators (ICDs), and cardiac resynchronisation therapy (CRT) devices are generating unprecedented volumes of data in both inpatient and remote settings. Artificial intelligence (AI) techniques are increasingly being applied to enhance the management of these devices and the patients who rely on them. Recent advances demonstrate that machine learning (ML) and deep learning (DL) can improve diagnostic capabilities (for example, by detecting arrhythmias and predicting clinical events), streamline remote monitoring workflows, and optimise device-based therapies. Key applications include AI-driven algorithms that accurately detect true arrhythmias while filtering out false alerts from pacemakers and implantable monitors, neural network models that predict ventricular arrhythmias weeks before ICD shocks, and personalised models that forecast which heart failure patients will respond to CRT. Moreover, novel approaches such as natural language processing (NLP) and reinforcement learning are being explored to integrate diverse data sources and to enable devices to self-adjust their programming. This narrative review summarises the major applications of AI in the CIED domain—diagnostics, remote monitoring, and therapy optimisation—with an emphasis on the recent literature over the past five years. The review highlights important studies and randomised trials in each area, discusses the variety of AI techniques employed, and outlines future directions and challenges (including data standardisation, validation in clinical trials, and regulatory considerations) for translating these innovations into routine clinical care.

## 1. Introduction

Implantable cardiac devices, including permanent pacemakers, intracardiac defibrillators (ICDs), cardiac resynchronisation therapy pacemaker (CRT-P), cardiac resynchronisation therapy defibrillator (CRT-D), as well as newer leadless pacemakers and subcutaneous ICDs, have become cornerstones of therapy for bradyarrhythmias, tachyarrhythmias, and heart failure (HF) [1]. Modern devices continuously record a wealth of physiological and technical data (heart rhythms, EGMs, device diagnostics, and sensor trends). The expansion of remote monitoring has further increased the frequency of device data transmissions, creating a deluge of information for clinicians to manage [2]. This explosion of CIED-generated data presents both an opportunity and a challenge: while rich in clinical insights, the volume and complexity can overwhelm traditional monitoring workflows [3]. Artificial intelligence (AI) has emerged as a promising tool to help unlock the value of this data while reducing burdens on healthcare providers [4,5]. Recent studies have begun to apply machine learning (ML) and deep learning algorithms to CIED data streams, aiming to enhance arrhythmia diagnostics, predict clinical decompensation, and automate or optimise device therapy settings [6].

This review provides a comprehensive overview of AI applications in the CIED domain, focusing on three major areas: (1) Diagnostics, where AI improves arrhythmia detection and risk stratification using device data; (2) Remote Monitoring, where AI algorithms triage incoming device alerts and assist in early identification of clinical issues; and (3) Therapy Optimization, where AI aids in tailoring device therapies like CRT and pacing to individual patient needs. Also, the review discusses the spectrum of AI techniques being utilised, from classical ML models to state-of-the-art deep learning, NLP, and even reinforcement learning, and highlights key findings from recent literature (particularly within the past five years). Notable randomised controlled trials and clinical studies are emphasised where available to underscore the evidence base. Finally, the review considers future directions, including the integration of multi-modal data, the need for prospective validation, and hurdles to clinical implementation.

### 1.1. Scientific Motivation for the Three Focus Areas

The review focuses on diagnostics, remote monitoring, and therapy optimisation because these represent the core clinical functions of cardiac implantable electronic devices and the areas where artificial intelligence has shown the strongest evidence of benefit. Diagnostics is central because implanted devices generate continuous intracardiac data that support arrhythmia detection and prediction. Remote monitoring has expanded rapidly and now produces high volumes of device alerts, making it a major driver for AI tools that improve triage, efficiency, and patient safety. Therapy optimisation is emerging as a key area because AI can refine patient selection, predict response to cardiac resynchronisation therapy, and support personalised device programming. Together, these three domains capture the full clinical value chain of implanted device care and identify the spaces where AI currently holds the greatest potential to improve outcomes.

### 1.2. Methodology

A structured process guided the identification and selection of relevant evidence. Identification involved electronic searches of PubMed and Google Scholar from January 2019 to January 2025 using predefined terms related to CIEDs, AI, diagnostics, monitoring, and therapy optimisation. Citation lists of key papers were also reviewed. Screening consisted of title and abstract assessment to remove studies not related to implantable devices, artificial intelligence, or clinically relevant applications. Eligibility was determined by reviewing the full text of remaining articles to exclude papers without original data, those focused solely on animal studies, or studies unrelated to device diagnostics, monitoring, or therapy optimisation. Inclusion involved selecting the final set of studies that met these criteria and contributed meaningful evidence about AI applications in CIEDs. This approach ensured comprehensive coverage while preserving the flexibility appropriate for a narrative review. The central research question guiding this review was How has AI been applied to cardiac implantable electronic devices to improve diagnostics, remote monitoring, and therapy optimisation, and what evidence supports its current and future clinical utility?

Although this is not a systematic review, elements of structured evidence appraisal were incorporated. The PICO framework was used to guide the search and selection process. PICO defines the Population, Intervention, Comparator, and Outcomes of interest. For this review, the population included patients with pacemakers, ICDs, CRT devices, leadless pacemakers, subcutaneous ICDs, and insertable cardiac monitors. Interventions included the application of artificial intelligence techniques to device diagnostics, monitoring workflows, and therapy optimisation. Comparators included standard device algorithms or conventional clinical assessment, where available. Outcomes included diagnostic accuracy, reduction in false alerts, arrhythmia prediction, workflow efficiency, CRT response prediction, infection risk, and device malfunction forecasting.

This structured approach supported transparent study selection while allowing flexibility appropriate for a narrative review. Formal quality scoring and meta-analysis were not performed.

### 1.3. Overview of Core AI Techniques Used in CIED Research

To support readers unfamiliar with technical terminology, this review briefly outlines the main AI methods applied in CIED-related studies. Supervised learning models, including logistic regression, random forests, gradient boosting, and neural networks, learn patterns from labelled datasets to classify events or make predictions such as arrhythmia risk or CRT response. Deep learning, particularly convolutional neural networks, is well-suited to analysing high-volume data streams such as EGMs and images. Natural language processing extracts structured meaning from clinical notes or device interrogation reports. Reinforcement learning is being explored for adaptive device programming based on continuous feedback. These methods differ in complexity but share the goal of identifying clinically relevant patterns within large, noisy device datasets.

## 2. AI in CIED Diagnostics

One of the earliest and most impactful applications of AI in this field has been improving cardiac arrhythmia detection and prediction using data from implanted devices. Pacemakers, ICDs, and insertable cardiac monitors continuously monitor patients and often issue alerts for events such as atrial fibrillation (AF) or ventricular tachycardia [5,7]. However, device algorithms can generate false-positive alerts or clinically insignificant findings, which can burden clinicians. AI techniques have shown the ability to refine these diagnostics. For example, integrating an AI-based filter into implantable loop recorders (ILRs) reduced false arrhythmia alerts by over 60%, markedly improving specificity for AF detections [8]. In a large real-world study, ILRs equipped with a deep learning algorithm (Medtronic’s AccuRhythm AI for AF and asystole detection) reduced non-actionable alerts by 58% compared to conventional ILRs, resulting in significantly fewer unnecessary transmissions and substantial workload savings [9]. These findings indicate that AI can help devices “decide” which events are truly important, so that clinicians are not inundated with benign data.

Beyond reducing false alerts, AI is being used to predict serious arrhythmic events before they occur. A landmark analysis by Ginder et al. applied machine learning to daily remote device data from ICD and CRT-D patients, successfully predicting appropriate ICD therapies (for ventricular tachyarrhythmias) up to 30 days in advance [10]. In that study, a neural network model achieved an area under the curve (AUC) of 0.90 for forecasting impending ventricular arrhythmias, significantly outperforming a traditional logistic regression model (AUC 0.72). The model identified subtle trends in device telemetry—such as changes in lead impedance, heart rate, and patient activity—that herald the onset of arrhythmia, illustrating how AI can extract prognostic signals invisible to human observers. Similarly, other groups have developed ML models to predict arrhythmic events, such as ICD shocks or ventricular tachycardia storms, using device data and patient characteristics [7,11]. These predictive algorithms could one day enable a proactive approach to device therapy, in which clinicians receive warnings of high-risk situations (an impending ventricular arrhythmia) and can intervene early.

AI has also enhanced the diagnostic interpretation of device-related data from other sources. A novel example is the application of computer vision to radiographic images: a neural network was trained to identify types of cardiac implantable devices on chest X-rays, accurately recognising the device manufacturer and model family, and exceeding the performance of expert humans [12]. This capability can be valuable in acute settings, for instance, when a patient with an unknown device presents to an emergency department. Additionally, natural language processing is being explored to analyse unstructured clinical notes and device interrogation reports to flag relevant diagnostic information. Although still nascent in this field, NLP could assist by extracting symptoms or contextual information surrounding device alerts (such as patient reports of syncope in device clinic notes) to help correlate device data with clinical status. Overall, AI-driven diagnostic enhancement, spanning signal processing, prediction models, and information extraction, is poised to increase the accuracy and utility of CIED detections while minimising noise.

### 2.1. Comparative Data Quality and AI Utility Across Monitoring Technologies

Data characteristics differ significantly between CIEDs, insertable cardiac monitors, and wearables. CIEDs provide continuous intracardiac signals with high fidelity, stable sensing vectors, and consistent beat detection. These characteristics support mature AI applications because noise levels are low, physiologic features remain stable over time, and device telemetry offers a reliable foundation for prediction models. Insertable cardiac monitors provide long-term rhythm surveillance but are subject to greater noise from motion and variable subcutaneous signal quality. AI tools for ICMs, therefore, focus mainly on filtering and alert refinement. Wearables provide a broad population reach but deliver the lowest signal quality, with higher susceptibility to artefacts and inconsistent data capture. AI applied to wearables remains useful for screening but offers lower precision than implantable systems.

**Figure 1:**
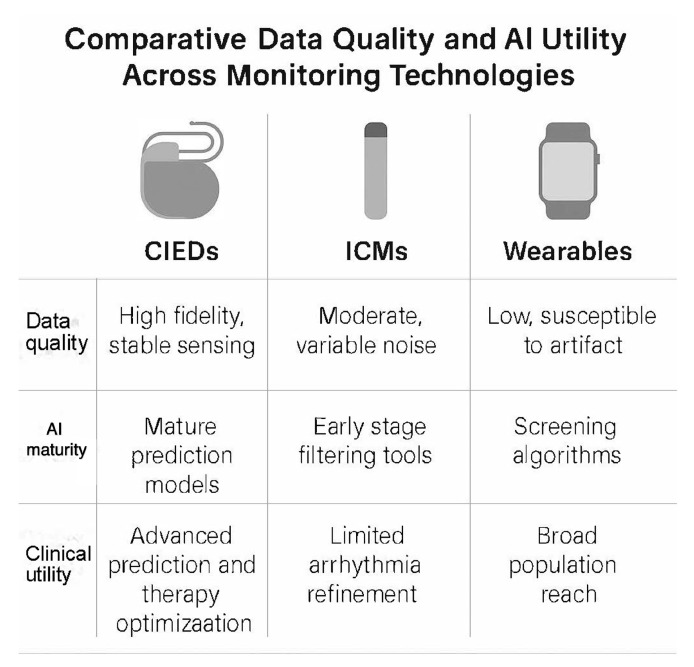
Comparative data quality and artificial intelligence utility across cardiac monitoring technologies. The figure contrasts key characteristics of cardiac implantable electronic devices, insertable cardiac monitors, and wearable sensors. CIEDs provide high-fidelity intracardiac data that support mature AI prediction models and advanced clinical applications such as therapy optimisation. ICMs offer moderate signal quality with variable noise, with AI mainly used for alert filtering and arrhythmia refinement. Wearables deliver low-fidelity surface signals with higher artefact susceptibility, while AI is primarily used for population-level screening. The comparison highlights why AI models achieve the highest clinical utility when built on continuous, high-quality data from implanted devices. ICM: intracardiac monitor. CIEDs: cardiac implantable electronic devices.

Across these three modalities, AI is most clinically actionable in devices that deliver high-quality, continuous data streams. CIEDs therefore provide a unique opportunity for advanced AI applications in arrhythmia prediction, risk stratification, and therapy optimisation. This distinction helps clarify the specific clinical value of AI within the implantable device ecosystem. Summary is demonstrated in Figure 1.

### 2.2. Clinical Context: Variability of AI-Based Arrhythmia Detection Across Patient Groups

AI performance in arrhythmia detection and monitoring may differ across cardiac phenotypes because each group exhibits distinct electrophysiologic patterns and device-derived trends. Patients with heart failure with reduced ejection fraction (HFrEF) often demonstrate higher burdens of ventricular arrhythmias, greater autonomic variability, and more pronounced changes in intracardiac signals before clinical deterioration. AI models trained on daily device diagnostics from these patients tend to rely on features such as heart rate variability, activity levels, and impedance trends that shift more abruptly during decompensation.

In contrast, heart failure with preserved ejection fraction (HFpEF) patients frequently present with atrial arrhythmias and less consistent ventricular substrate abnormalities. Atrial fibrillation alerts in this population often generate a higher proportion of false positives due to baseline ectopy and intermittent conduction variability. AI filters applied to ILRs and pacemaker-based AF diagnostics provide greater incremental benefit in improving specificity for HFpEF patients.

Other phenotypes, including cardiomyopathies, congenital heart disease, and infiltrative disorders, exhibit unique EGMs patterns or lead behaviour that influence AI classification. For example, fibrotic substrates in hypertrophic or dilated cardiomyopathy alter sensing vectors and may introduce noise, requiring model recalibration. Patients with congenital heart disease often have non-standard anatomy or pacing configurations, which can challenge generic prediction models.

Recognising these phenotypic differences is important when developing or deploying AI models, since training on heterogeneous populations may dilute performance unless stratified learning or phenotype-aware calibration is incorporated. As device manufacturers increasingly integrate AI algorithms, phenotype-specific optimisation may enhance clinical accuracy and reduce alert burden.

## 3. AI for Remote Monitoring and Data Management

Remote monitoring (RM) of CIEDs has become standard of care, enabling devices to transmit alerts and data to clinicians between in-office visits [13]. Evidence from multiple trials has shown that RM is as effective as (or superior to) traditional in-clinic follow-ups in detecting problems early, reducing inappropriate shocks, and even improving clinical outcomes like mortality [6]. However, along with its benefits, RM greatly increases the volume of device data that clinics must process daily [2]. AI-based solutions are now being leveraged to handle this “data deluge” and streamline RM workflows. The central idea is to use algorithms to triage incoming transmissions, so that truly urgent issues are promptly flagged to physicians. At the same time, benign or low-priority alerts are filtered or handled automatically.

**Figure 2:**
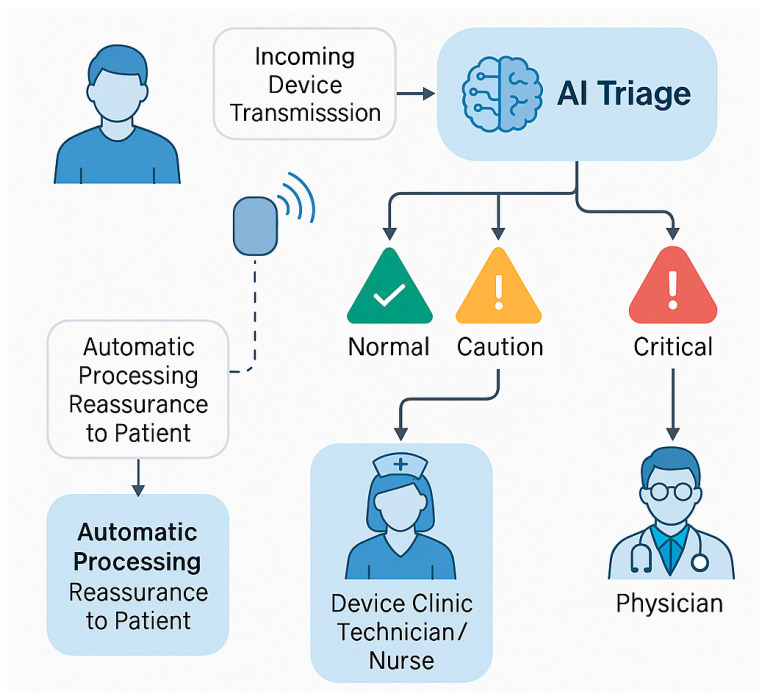
Schematic representation of an artificial intelligence (AI)-enhanced remote monitoring system for patients with cardiac implantable electronic devices (CIEDs). Incoming device transmissions are automatically triaged by an AI algorithm, which classifies alerts into three urgency tiers: normal, caution, and critical. Transmissions deemed normal (i.e., no abnormalities detected) are automatically processed, potentially providing immediate reassurance to patients without requiring clinician input. Alerts classified as caution or critical are escalated through a structured review process—first to device clinic technicians or nurses, and subsequently to physicians for evaluation and intervention. This AI-driven triage significantly reduces the volume of non-actionable alerts, improves clinical efficiency, and ensures timely response to significant device-related events such as arrhythmias or malfunctions.

Recent studies have demonstrated impressive gains in efficiency through AI-assisted remote monitoring. Rosier and colleagues, for example, showed that a customised system combining pacemaker alert data with patients’ clinical histories could automatically triage alerts and reduce the notification workload by 84% [14]. More recently, Bawa et al. reported on an AI-driven triage algorithm that processes CIED remote transmissions and categorises them as “safe,” “caution,” or “critical,” effectively creating a three-tier review system [15]. In this model, only caution and critical alerts require human evaluation, whereas safe and normal transmissions can be automatically adjudicated, and the patient can be given reassurance without clinician input. Such an approach was projected to dramatically reduce the number of transmissions requiring manual review, allowing staff to focus on the small fraction of cases with potential abnormalities [16]. Early implementations of these systems have yielded tangible benefits—for instance, a multicentre analysis found that clinics using AI-enhanced ILRs experienced a significant reduction in total alert volume and saved an estimated 559 personnel hours per year for every 600 patients on remote monitoring [9]. Figure 2 illustrates a schematic overview of an AI-assisted remote monitoring workflow for CIEDs, showing the automated categorisation of device alerts into normal, caution, and critical categories, along with corresponding clinical escalation. By filtering out false alarms and routine data, such AI-driven triage systems markedly reduce the workload on clinicians while ensuring that urgent events (for example, a serious arrhythmia or device malfunction) are promptly addressed [2].

Another domain where remote monitoring, where AI has shown promise, is in the early detection of clinical deterioration. CIEDs in heart failure patients often track physiological parameters (heart rate trends, patient activity, thoracic impedance, etc.) that can serve as early warning signs of heart failure exacerbation. AI algorithms can integrate these multi-parameter data to forecast decompensation before the patient becomes overtly symptomatic. Ongoing research is exploring machine learning models to interpret device-based heart failure alerts (rapid weight gain, rising impedance indicating fluid retention) with fewer false positives [17]. The goal is to use AI to distinguish meaningful heart failure alerts from noise, so that clinicians can be notified of patients truly at risk of hospitalisation and intervene promptly (for example, adjust diuretics). Although robust AI solutions for heart failure monitoring via CIEDs are still under development, preliminary efforts indicate improved specificity in identifying patients who need clinical action [18]. Future large-scale validation will be required, but AI could become an integral part of remote device monitoring platforms, functioning as a smart filter and decision support tool that ensures the right information reaches providers at the right time.

## 4. AI in Therapy Optimisation and Device Programming

The most forward-looking applications of AI in the CIED realm involve optimising the delivery of device therapies. This encompasses both selecting the right patients for device-based therapies and fine-tuning device settings to maximise benefit. A prime example is CRT, in which only a subset of heart failure patients responds robustly despite meeting standard selection criteria. In recent years, researchers have applied AI to improve patient selection and customisation of CRT. Supervised and unsupervised ML models have been trained on data from major CRT trials to identify predictors of CRT response [19]. Some models have incorporated dozens of clinical and echocardiographic variables. In contrast, others have used DL on raw ECG or imaging data to find subtle features that signal a positive CRT response [20,21]. Notably, a simpler model using a naïve Bayes classifier with nine clinical variables was developed and validated to predict CRT outcomes, leading to a publicly available calculator intended to aid shared decision-making for CRT candidates [19,22]. Such AI-derived tools can supplement conventional criteria (like QRS duration and morphology) by providing an individualised probability of response, potentially improving the selection of patients most likely to benefit from CRT.

Beyond predicting who will respond, AI is also being used to optimise how therapy is delivered. In CRT, the precise positioning of leads and programming of timing intervals (AV and VV delays) are critical to success. Advanced imaging techniques, combined with AI analysis, are now being investigated to guide lead placement [23,24]. By training on imaging and outcomes from hundreds of patients, the AI can learn to recognise patterns of scar or mechanical dyssynchrony that a human operator might miss, ultimately suggesting how to tailor the CRT approach for each patient. Early results have been promising, and efforts are underway to translate these algorithms into software that clinicians can use for planning CRT procedures [25]. In the realm of conventional pacemakers, AI might guide programming for rate responsiveness or manage complex pacing strategies such as His-bundle or left bundle branch area pacing by analysing ECG feedback in real time (though these applications remain largely investigational).

Another frontier is the use of reinforcement learning (RL) for autonomous device programming. RL is a type of AI where an algorithm learns to make optimal decisions through trial-and-error interactions with its environment. In the context of CIEDs, an RL-based controller could, in theory, continuously adjust a device’s settings, “learning” the optimal programming for a given patient’s condition [26]. For example, an RL algorithm in a pacemaker could tweak the timing of pacing pulses or sensor thresholds in response to the patient’s activity levels and feedback on cardiac output, to maximise hemodynamic performance. The key advantage is personalisation: the device adapts to the individual rather than relying on one-size-fits-all programming. A recent review by Komp et al. discussed strategies for safe reinforcement learning in CIED programming, emphasising the need to balance exploration (trying new settings to seek improvement) against exploitation (using settings known to be effective) [26]. Unconstrained exploration by an algorithm can pose risks if a harmful setting is applied; therefore, safety-focused designs (for instance, sandbox testing of new parameters and predefined safety limits) are crucial. While RL in implanted devices is still theoretical, preliminary simulations suggest that closed-loop AI control could one day react faster than clinicians to a patient’s changing condition and continuously optimise therapy. Table 1 summarises major AI applications in CIEDs across diagnostics, monitoring, and therapy optimisation, along with examples of key findings from recent studies.

## 5. AI in Predicting CIED Infections and Device Malfunctions

Predicting CIED Infections: CIED infections occur in approximately 1–2% of implants and are associated with high morbidity, mortality, and cost [27]. Given this burden, recent efforts have applied AI and advanced analytics to identify patients at risk. Traditional risk stratification has relied on known clinical factors (e.g., renal dysfunction, reoperations, procedure duration) mostly from retrospective studies, with only one prospective trial to date. Now, machine learning is being used to integrate these factors into more accurate prediction tools. Conceptual workflow: Example of an automated tool leveraging electronic health record data to flag potential CIED infections for targeted prevention efforts [28]. A notable development is the BLISTER score; a risk model derived through multivariate analysis of more than 7000 CIED procedures. BLISTER incorporates variables such as elevated peri-procedural C-reactive protein, prolonged procedure time (>120 min), recent re-interventions, immunosuppression, renal function, and prior procedures [29]. In an external validation study, BLISTER achieved an AUC of 0.80 for 12-month infection, outperforming the older PADIT score (AUC ~0.75). Importantly, applying BLISTER in decision modelling showed that using an antibiotic envelope only in high-risk patients (score ≥ 6, ~18% of patients) could cut 1-year infection rates from ~1.0% to 0.7% (relative risk reduction ~30%) [29]. This targeted strategy was cost-effective, yielding an incremental cost per QALY of approximately £18,400—well within typical acceptable thresholds. These findings suggest that AI-driven risk scores can facilitate cost-effective prophylaxis (envelopes) by sparing low-risk patients. Beyond risk scores, natural language processing and EHR-based algorithms have been explored to detect incipient infections. For example, a Veterans Affairs study combined structured alerts (such as microbiology orders and antibiotic prescriptions) with text mining of clinical notes to flag CIED infections, achieving a c-statistic of ~0.95 with a sensitivity of ~88% [28]. While such tools focus on early identification rather than prediction, they illustrate the power of AI to survey large datasets for infection signals. Overall, AI models for CIED infection risk show promise for stratifying patients to enhance preventive measures. Key limitations include reliance on retrospective data with low event rates (~1% infections), potential overfitting, and the need for prospective validation. No randomised trials have yet tested an “AI-guided” infection prevention strategy, though the demonstrated accuracy and cost-utility benefits support future clinical implementation. Experts also note that remote monitoring data from CIEDs remains an underused resource for infection surveillance. In this area, future AI applications (detecting early physiological or impedance changes) could further improve the timeliness of intervention.

Predicting Device Malfunctions: AI is likewise being leveraged to predict CIED hardware or therapeutic malfunctions before they occur. This includes forecasting battery depletion, lead failures, and inappropriate or failed therapies. Traditionally, device management relies on threshold alarms (e.g., impedance alerts) and periodic checks; AI can augment this by recognising complex multi-parameter patterns. A recent study applied machine learning to pacemaker interrogation data to predict battery longevity, using features such as battery voltage, lead impedances, pacing burden, and capture thresholds [30]. A neural network model achieved near-perfect accuracy (R^2^ ≈ 1.0) in forecasting remaining battery life (mean error < 0.1 months), far outpacing linear regression methods [30]. Such a tool could enable proactive generator replacements and prevent unexpected depletion, integrating with remote follow-up systems to trigger automated alerts. In the realm of ICDs, researchers have trained AI to predict inappropriate shocks and other therapy issues. For instance, Tateishi et al. (2023) [31] developed machine-learning classifiers (testing 14 algorithms) on post-implant clinical and ECG data to identify patients likely to receive inappropriate ICD therapy (e.g., shocks for non-lethal arrhythmias). The best model (an extra-trees ensemble) achieved an AUC of 0.87 in held-out data [31]. From this, they derived a simplified risk score (“Cardi35”) based on six key predictors (e.g., history of atrial arrhythmias, absence of CRT, certain ECG features), which showed that high scores were associated with a hazard ratio of ~1.6 for inappropriate shocks [31]. This demonstrates how AI can uncover novel risk combinations (notably, the model identified the absence of diabetes as increasing shock risk) and translate them into a usable score [31]. Similarly, others have mined device telemetric data to predict impending ICD therapies. Using continuous remote monitoring feeds from ICDs, one group built models (random forests, XGBoost, one-dimensional convolutional neural network [1-D CNN]) to predict shocks or anti-tachycardia pacing up to 4–30 days before they occurred [32]. The optimal model (4-day random forest) achieved an AUC of ~0.86 and 84% accuracy in forecasting imminent therapy needs [32]. Notably, the most predictive features were device-recorded: frequent non-sustained ventricular tachycardia episodes, prior therapies, patient activity trends, lead parameters, and mean heart rate variability. These examples demonstrate that AI can process complex, high-volume data from CIED diagnostics to warn of potential failures or arrhythmic events before they manifest clinically. However, these studies remain at a developmental stage. Sample sizes are modest (in the hundreds of patients), and models may be site-specific. Prospective trials are lacking—it remains to be shown that AI predictions can be reliably translated into clinical action (e.g., early lead revision or reprogramming) that improves outcomes. Additionally, regulatory considerations for algorithms that influence device management are non-trivial, given the imperative of patient safety. Despite these challenges, emerging evidence suggests that AI-driven predictive maintenance for CIEDs could enhance device reliability and patient safety, thereby reducing the risk of sudden malfunctions and the need for emergency interventions [33].

## 6. Economic and Health System Implications of AI in CIED

The integration of AI into CIED care has significant economic and system-level ramifications. Broadly, AI-enabled strategies (such as remote monitoring with automated analytics and risk-guided interventions) promise to improve efficiency, reduce adverse event costs, and reshape clinical workflows. One of the most mature applications is remote monitoring (RM) of pacemakers and ICDs, whereby device diagnostics are transmitted electronically and reviewed off-site. AI can augment RM by triaging large data streams and prioritising clinically significant alerts, thereby streamlining workflow and reducing alarm fatigue among clinicians [32]. Indeed, even without sophisticated AI, RM itself has been shown to reduce in-person follow-ups and enable earlier issue detection. Economic evaluations over the past decade have increasingly demonstrated the cost benefits of RM for CIED patients. A 2023 systematic review compiled 27 studies comparing RM with conventional clinic follow-up, finding that 78% of studies reported RM was associated with net cost savings to the health system [34]. Several analyses noted that remote follow-ups reduce travel and hospital visit costs, expedite interventions for technical or clinical issues, and prevent costly emergency admissions (for example, by early detection of lead problems or arrhythmias). Moreover, among 13 formal economic evaluations in that review, all six cost–utility analyses agreed that RM provides additional quality-adjusted life years (QALYs) at an acceptable incremental cost, meeting conventional thresholds for cost-effectiveness in healthcare [34]. In summary, RM of CIEDs—often considered an early foray of “AI” in device care—is generally cost-saving or cost-effective across different health systems. This aligns with real-world experience during the COVID-19 era, where remote device management maintained care quality while reducing clinic burden.

AI’s impact extends to resource utilisation and clinical workload. By automating data analysis, AI can reduce the labour of routine device checks and focus human attention on high-risk cases. For example, ML algorithms that automatically flag patients at an elevated risk of infection or device malfunction enable the targeted scheduling of prophylactic measures or early interventions, rather than one-size-fits-all monitoring. This targeted approach can translate into more efficient use of hospital resources—e.g., only ~18% of patients might need an expensive antibacterial device envelope if guided by an AI risk score, as opposed to blanket usage [29]. Such targeting yields better value: the BLISTER score’s selective envelope strategy was shown to remain under £20,000 per QALY, whereas universal envelope use would likely exceed typical cost-effectiveness thresholds [29]. Likewise, AI-driven remote monitoring can decrease unnecessary clinic visits (saving staff time and patient time) and reduce unscheduled emergency presentations by anticipating issues. A Canadian analysis found that a single CIED infection hospitalisation averages ~$50–77,000 in costs (depending on the interventions required) [35], much of which is driven by prolonged hospital stays and complex procedures. Preventing even a handful of such infections via AI-informed prophylaxis can offset substantial costs. European data similarly show that CIED infections often incur unreimbursed costs, amounting to financial losses for hospitals in many cases, underscoring the economic imperative for smarter prevention [27]. By reducing adverse events, AI could thus alleviate strain on hospital budgets and bed capacity.

Formal health economic evaluations of AI tools in CIED care are still limited but are emerging. Some focus on preventive technologies: for instance, cost-effectiveness models in Denmark and Canada evaluated the adjunctive antibiotic envelope using risk-based approaches. In Denmark, applying envelopes in high-risk CRT patients undergoing generator replacement was deemed cost-effective, considering the high baseline infection risk in this subgroup [36]. A parallel analysis from the US concluded that the absorbable envelope could be economically justified when factoring in infection-related costs and improved patient survival [37]. These studies reinforce that targeted use of novel technologies (guided by risk stratification algorithms) can yield favourable economic outcomes. Another angle is the impact on clinical workflow and staffing. Suppose AI can automate routine data review (for example, filtering out benign device alerts). In that case, electrophysiology clinics might manage larger CIED populations without proportional increases in staff or reallocate specialist time to more complex decision-making. Some reports have highlighted that current adoption of remote device management remains suboptimal in part due to reimbursement and funding barriers [34]. Demonstrating clear cost-saving through AI and securing payment models for remote/AI services will be key to broader implementation. Importantly, the upfront costs of AI integration (including software, training, and data infrastructure) must be weighed against the downstream savings. While many AI interventions show positive returns in modelling, real-world deployment needs monitoring for any unintended costs—e.g., responding to false-positive alerts or system maintenance.

**Figure 3:**
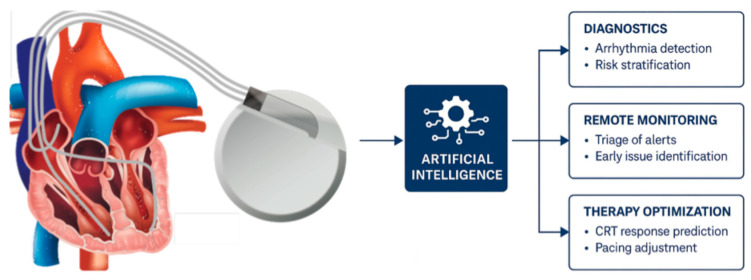
Artificial intelligence applications in cardiac implantable electronic devices. AI integration enhances device utility across three domains: diagnostics (arrhythmia detection and risk stratification), remote monitoring (triage of alerts and early issue identification), and therapy optimisation (cardiac resynchronisation therapy response prediction and pacing adjustment).

In summary, the use of AI in CIED management appears to enhance value-based care: improving outcomes (fewer infections, fewer emergency shocks) while optimising resource use. Remote monitoring augmented by AI has repeatedly shown cost savings and efficiency gains in recent studies. Risk-prediction models enable precision medicine approaches (focusing expensive preventive or therapeutic resources on the patients who benefit most) with favourable cost–benefit profiles. Though dedicated randomised trials of “AI-guided” CIED care are still lacking, current evidence suggests that thoughtfully implemented AI can reduce health system costs, lighten clinician workload, and improve patient safety. Ongoing health economic evaluations and pilot programs will further clarify the return on investment for AI in this space. If these positive trends continue, one can anticipate wider adoption of AI-driven protocols in electrophysiology practice, supported by policies that reimburse digital and AI-enabled services in device care. Ultimately, aligning AI innovation with cost-effectiveness and workflow efficiency will be crucial for the sustainable integration of AI into CIED clinical practice (Figure 3).

## 7. Future Directions and Challenges

The integration of AI into CIED management is advancing rapidly; however, several important steps are needed to transition current innovations from research to routine clinical practice. One priority is prospective validation of AI tools through clinical trials. Many of the algorithms discussed (arrhythmia prediction models or AI-guided CRT decision tools) have demonstrated impressive performance in retrospective or observational studies [25]. The next step will be to test whether using these AI tools in real-time patient care improves outcomes, as has already been shown in other cardiac conditions, including AF [38]. This may involve randomised controlled trials in which one group of patients is managed with AI-assisted device monitoring or programming and compared with standard care. Early randomised studies in cardiology have highlighted both the potential and pitfalls of AI [39]. For instance, some AI-driven diagnostic tools have improved early disease detection, but others have failed to affect hard clinical endpoints [40]. Thus, rigorous evaluation in the CIED context will be crucial to identify which AI applications truly add value for patients (reducing arrhythmia events, preventing hospitalisations, or improving survival).

Another challenge is the heterogeneity of data and systems. CIEDs from different manufacturers output data in proprietary formats and use distinct algorithms for event detection. AI systems will need to interface with multiple device ecosystems and possibly aggregate data across them. Efforts in data standardization. For example, common data models for device diagnostics and a unified platform for remote monitoring data could greatly facilitate the development of robust, generalizable AI solutions [2]. Collaborative initiatives between device companies, clinicians, and data scientists might be necessary to curate large, representative datasets for AI training and validation. Privacy and data security are additional considerations, since remote device data and electronic health records contain sensitive patient information [41]. Ensuring compliance with data protection regulations is mandatory when deploying AI in this space, especially when using cloud-based analytics.

On the technical front, improving the transparency and interpretability of AI algorithms will increase clinician trust in these tools. Black-box models, particularly deep learning networks, can be highly accurate but often lack explainability. In critical domains, such as device therapy (where an incorrect decision could result in patient harm), physicians are likely to demand clear reasoning or evidence behind an AI’s recommendations [42]. Techniques for explainable AI, such as algorithms that highlight which data features influenced a prediction (lead impedance trend, heart rate variability change), could help users validate and embrace AI outputs. Additionally, building user-friendly interfaces into device clinic workflows is important. The ideal scenario is an AI that runs in the background of a device remote monitoring system or programmer software, giving gentle decision support prompts (for example, “this patient’s transmission is likely a false alarm” or “consider reprogramming this setting based on patient profile”) without interrupting the clinician’s normal workflow (Figure 2).

Ultimately, regulatory pathways for AI in medical devices are undergoing evolution. AI algorithms that directly influence CIED function (such as an autonomous RL-based pacing algorithm) are high-risk and require thorough pre-market evaluation and, if approved, post-market surveillance. It will be essential for developers to collaborate closely with regulators and adhere to frameworks to ensure that these algorithms maintain consistent performance across diverse patient populations and device hardware/software updates. The prospect of in vivo learning systems (devices that continuously modify their behaviour via AI) is especially challenging from a regulatory standpoint and may require novel oversight mechanisms.

Despite these challenges, the momentum behind AI in cardiology suggests that many of these hurdles will be overcome. International societies and expert consensus statements are beginning to outline best practices for digital innovations in arrhythmia care [43]. As positive clinical experience accumulates, clinicians can expect AI-driven features to be increasingly incorporated into next-generation CIEDs and their associated monitoring infrastructure.

## 8. Conclusions

AI is poised to significantly augment the capabilities of cardiac implantable electronic devices, ushering in a new era of “smart” pacemakers and defibrillators. In diagnostics, AI algorithms can enhance the accuracy of arrhythmia detection and even forecast dangerous events before they occur. In the arena of remote monitoring, AI is already proving its value by sifting through floods of device data to focus clinicians’ attention on the patients who need it most, thereby improving efficiency and potentially patient outcomes. For therapy optimisation, AI offers the promise of truly personalised device therapy, from selecting the right patients and ideal device type, to optimising lead placement and device programming in a data-driven manner. Importantly, these advances have been driven by a range of AI techniques (machine learning models, deep neural networks, NLP, reinforcement learning, etc.), each chosen to suit specific tasks within the CIED ecosystem.

The literature of the past five years highlights both the excitement and the work ahead. Early clinical studies and randomised trials indicate that AI-supported care can reduce unnecessary shocks, clinic visits, and workloads, while improving diagnostic yield. However, more evidence from large trials is needed to establish the benefits of patient-centric outcomes firmly. As AI continues to evolve, close collaboration between clinicians, engineers, and regulators will be essential to ensure these technologies are safe, effective, and equitable. In the foreseeable future, cardiologists and electrophysiologists will likely interact with AI-generated insights, such as device algorithm alerts or recommendations from a decision support tool, as part of standard device management. By embracing and refining these tools through real-world experience, the community can harness AI’s potential to improve the quality of patient care for those with CIEDs. Ultimately, integrating AI into cardiac implantable devices holds great promise for enhancing diagnostic precision, patient safety, and therapeutic outcomes in cardiology.

## Figures and Tables

**Table 1:** Major applications of AI in the CIED domain, with examples from recent studies. AI approaches range from classical machine learning (e.g., logistic regression, random forests) to deep learning neural networks, and even reinforcement learning for autonomous optimisation. These techniques are being applied to improve arrhythmia diagnostics, manage the influx of remote monitoring data, and tailor device therapies to individual patients.

Application	AI Approach	Example Findings
Arrhythmia detection and prediction	ML/DL on device data streams	Deep neural networks analysing daily device telemetry predicted ICD shocks up to 30 days early (AUC ~0.90). AI algorithms for insertable monitors cut false arrhythmia alerts by >60%.
Remote monitoring alert triage	Automated filtering (rule-based + ML)	Integrated data systems reduced device alert workload by ~84%. AI-enhanced monitors (with smart filters) achieved ~58% fewer non-actionable alerts, saving ~559 staff hours annually per clinic.
Therapy optimization (CRT)	Predictive modelling; imaging analysis	ML models trained on CRT trial data can predict responders (one 9-variable model is available as an online tool). AI-guided MRI analysis is being used to plan optimal lead placement for CRT.
Device programming automation	Reinforcement learning control	Proposed safe-RL frameworks allow pacemakers/ICDs to self-adjust settings based on patient physiology, while using safeguards to prevent unsafe changes.

## Data Availability

No new data were created or analysed in this study. Data sharing is not applicable to this article.

## Abbreviations

AF, atrial fibrillation; AI, artificial intelligence; AUC, area under the receiver operating characteristic curve; AV, atrioventricular; CIED, cardiac implantable electronic device; CRT, cardiac resynchronisation therapy; CRT-D, cardiac resynchronisation therapy defibrillator; CRT-P, cardiac resynchronisation therapy pacemaker; CT, computed tomography; DL, deep learning; ECG, electrocardiogram; EHR, electronic health record; HF, heart failure; ICD, implantable cardioverter-defibrillator; ILR, insertable loop recorder; ML, machine learning; MRI, magnetic resonance imaging; NIHR, National Institute for Health and Care Research; NLP, natural language processing; QALY, quality-adjusted life year; QRS, QRS complex; RL, reinforcement learning; RM, remote monitoring.
